# Species Boundaries and Parapatric Speciation in the Complex of Alpine Shrubs, *Rosa sericea* (Rosaceae), Based on Population Genetics and Ecological Tolerances

**DOI:** 10.3389/fpls.2019.00321

**Published:** 2019-03-18

**Authors:** Yun-Dong Gao, Xin-Fen Gao, Aj Harris

**Affiliations:** ^1^CAS Key Laboratory of Mountain Ecological Restoration and Bioresource Utilization and Ecological Restoration and Biodiversity Conservation Key Laboratory of Sichuan Province, Chengdu Institute of Biology, Chinese Academy of Sciences, Chengdu, China; ^2^Oberlin College and Conservatory, Department of Biology, Oberlin, OH, United States

**Keywords:** biodiversity, eastern Himalayas, ecological niche modeling, ecological speciation, population genetics, species concept

## Abstract

Discerning species boundaries among closely related taxa is fundamental to studying evolution and biodiversity. However, species boundaries can be difficult to access in plants because ongoing divergence and speciation may leave an evolutionary footprint similar to introgression, which occurs frequently among species and genera. In this study, we sought to determine species boundaries between two closely related alpine shrubs, *Rosa sericea* and *Rosa omeiensis*, using population genetics, environmental data and ecological niche modeling, and morphological traits. We analyzed populations of *R. sericea* and *R. omeiensis* using genetic markers comprising a fragment of the single-copy nuclear gene, LEAFY, micro-satellites (EST-SSR), and plastid DNA sequences. The DNA sequence data suggested clusters of populations consistent with geography but not with previously proposed species boundaries based on morphology. Nevertheless, we found that the ecological niches of the previously proposed species only partially overlap. Thus, we suspect that these species are in the process of parapatric speciation; that is, differentiating along an ecological gradient, so that they exhibit differing morphology. Morphology has previously been the basis of recognizing the species *R.* *sericea* and *R. omeiensis*, which are the most widely distributed species within a broader *R. sericea* complex that includes several other narrow endemics. Here, we recognize *R.* *sericea* and *R. omeiensis* as independent species based on morphological and ecological data under the unified species concept, which emphasizes that these data types are of equal value to DNA for determining species boundaries and refining taxonomic treatments. While the DNA data did not delimit species within the *R.* *sericea* complex, we expect to develop and utilize new, robust DNA tools for understanding speciation within this group in future studies.

## Introduction

Species comprise a basic unit of biology and are fundamental to elucidating first order patterns in the organization of global, regional and local biodiversity, such as the latitudinal gradient of species richness (von Humboldt, 1807; Willig et al., 2003; Mannion et al., 2014), Asian species bias among eastern Asian-eastern North American disjunct genera (Wen, 1999, 2001; Qian and Ricklefs, 2000), and the species-area relationship (Arrhenius, 1921). Additionally, species are at the heart of calls to document and conserve existing biodiversity in the face of the extinction crisis of the Anthropocene (Wilson, 1988; Agapow et al., 2004; Ceballos et al., 2015).

Equally important to delimiting boundaries among species for all aspects of biodiversity science, is the philosophy that we apply to delimitation; that is the species concept. Despite the centrality of species to understanding biology, biodiversity science, and conservation, delimiting species from one another remains a controversial exercise for biologists, so that dozens of species concept have been raised (De Queiroz, 2005, 2007; Hey, 2006; Freudenstein et al., 2017). For example, in plants alone, authors have debated the merits of an evolutionary species concept (George, 1951), which emphasizes monophyletic lineages, versus a biological one (Mayr, 1942), which emphasizes reproductive isolation as the key and ultimate standard for recognizing species (Coyne and Orr, 2004) reproductive isolation as the key and ultimate standard for recognizing species. Overall, many species concepts are narrow; focusing on one or a few aspects of reproductive isolation, such as genetic or morphological differences (De Queiroz, 2007; Aldhebiani, 2018). These narrow concepts are insufficient, because evolution is ongoing such that divergence may be incomplete in the present [e.g., *Orinus* Hitchc. (Liu et al., 2018), see also (Funk and Omland, 2003; Arnold, 2016)] and may affect genetics, morphology, ecology, and other aspects of plant biology at different rates. Moreover, species delimitation is frequently confounded in plants during secondary contact that can lead to introgression and hybridization even after 10s of millions of years of evolution in isolation (e.g., in buckeyes; Hardin, 1957; Harris et al., 2009; Aldhebiani, 2018). Therefore, recent species concepts, such as the unified species concept (De Queiroz, 2005, 2007), seek holistic approaches to species delimitation to accommodate these and other complexities of evolutionary divergence. The unified species concept integrates over all of the available kinds of data such as morphology, molecular sequences, and ecology (De Queiroz, 2007). Like the ‘integrative species concept’ (Liu, 2016), the unified species concept aims to strike a balance among rival concepts and provide a practical philosophy of species delimitation.

The unified species concept is especially useful for delimiting species that arise from recent or ongoing parapatric speciation in contrast to biological species concepts, which tend to assume an underlying allopatric speciation model (Mayr, 1942; Coyne and Orr, 2004). In plants, parapatric speciation, or diversification along an environmental gradient, probably occurs commonly (Gavrilets et al., 2000; Stuart et al., 2006; Florio et al., 2012). However, detecting parapatric speciation can be challenging because it may frequently be driven by the same mechanisms that drive other evolutionary modes, such as allopatric speciation and secondary contact leading to introgression. These mechanisms include mountain uplift, climatic perturbations, or, more broadly, ecological disturbance (Colin and Guy, 2015; Zhao et al., 2016). Nevertheless, parapatric speciation in plants is particularly widely hypothesized to occur along elevational gradients (Watson and Spottiswoode, 1835; Bond, 1989; Knox and Palmer, 1995; Carpenter, 2005; Givnish et al., 2009; Sanders and Rahbek, 2012; Caro et al., 2013; Guo et al., 2013), which co-vary with other environmental factors of importance to plants such as temperature, precipitation, and light availability (e.g., Qian and Sandel, 2017). During and immediately following parapatric speciation, species boundaries may be difficult to ascertain using molecular data due to recency of divergence and the lack of strict geographic barriers to gene flow (Abbott and Brennan, 2014). Moreover, morphological differences between the new or emerging species may (Harrison, 2012; Harrison and Larson, 2014) or may not be detectable (Stuart et al., 2006; Florio et al., 2012). However, thorough integration of morphological, molecular, and ecological data may help to discern parapatric speciation from among other evolutionary processes, determine species boundaries, and provide the basis for application of a unified species concept of taxonomic revisions.

Within the mountainous region of southwestern China, including the Himalayas and the Hengduan Mountains, many recent environmental perturbations, such as uplift, glaciation, and shifts in the monsoon climate, represent possible drivers of speciation and, consequently, explanations for the vast botanical biodiversity of the region (Myers et al., 2000; An et al., 2001; Wharton et al., 2005; Ju et al., 2007; Xing and Ree, 2017). For example, uplift within the Hengduan Mountains 3–4 million years (m.y.) ago (Chen, 1992, 1996) may have presented new ecological opportunities for plants (and animals) along emerging elevational gradients (e.g., Liu et al., 2006; Gao et al., 2013, 2015a; Xing and Ree, 2017). Similarly, glacial cycles (0.9–2.4 m.y. ago) during the Quaternary also resulted in shifting vegetational patterns along elevational gradients as well as isolation within refugia and subsequent secondary contact (Williams et al., 1998; Hewitt, 2000). Therefore, the mountains of southwestern China represent an area ripe for parapatric speciation events, though these are likely to have co-occurred with other modes of speciation.

**FIGURE 1:**
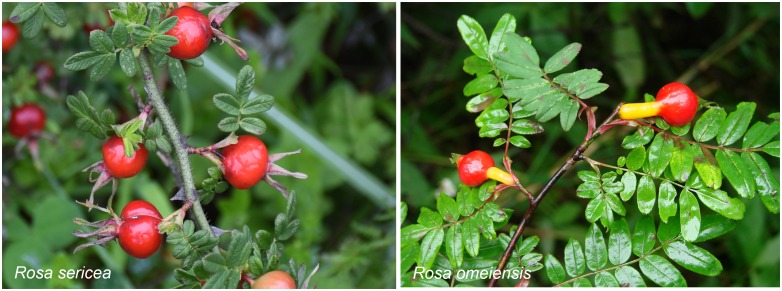
Representative individuals of *Rosa sericea* and *Rosa omeiensis* showing their morphological differences according to their treatment in the Flora of China. Images show individuals at roughly the same stage of fruit development.

Here, we investigate the evolutionary mechanism of speciation within two well-recognized sister species that are endemic to the mountains of southwestern China (Gao et al., 2015b): *Rosa sericea* Lindley and *Rosa omeiensis* Rolfe. Both species occur along a high latitudinal gradient from 1000 to 4000 m above sea level within the Qinghai-Tibetan Plateau (QTP), the Hengduan Mountains, and adjacent uplands. *Rosa sericea* comprises understory plants within forested areas while *R. omeiensis* occurs above the tree line as an alpine shrub. The two species have large populations and relatively distinct morphologies, which intergrade within parts of the geographic range. *Rosa sericea* is distinguished from *R. omeiensis* by having fewer leaflets (7–11 compared to 9–17) and by an obovoid or globose hip with slim pedicle (compared to an obovoid or pyriform hip, with a conspicuous, stout, tapering pedicel, Figure 1; Ku, 1985; Ku and Robertson, 2003). However, intergrading morphology has made delimitation of these species challenging (Ku, 1985; Ku and Robertson, 2003; Fang et al., 2011; Gao et al., 2015b).

We hypothesized that *R. sericea* and *R. omeiensis* represent independent species based on morphology and that they have diverged or are diverging along an altitudinal gradient consistent with our observations in the field. Therefore, we applied population genetics and morphological and ecological data to attain the following objectives: (1) assess species boundaries between *R. sericea* and *R. omeiensis*; (2) evaluate gene flow and divergence between the species and determine the mode of speciation if any; and (3) discuss our results within the context of species concepts.

## Materials and Methods

### Plant Sampling

**FIGURE 2:**
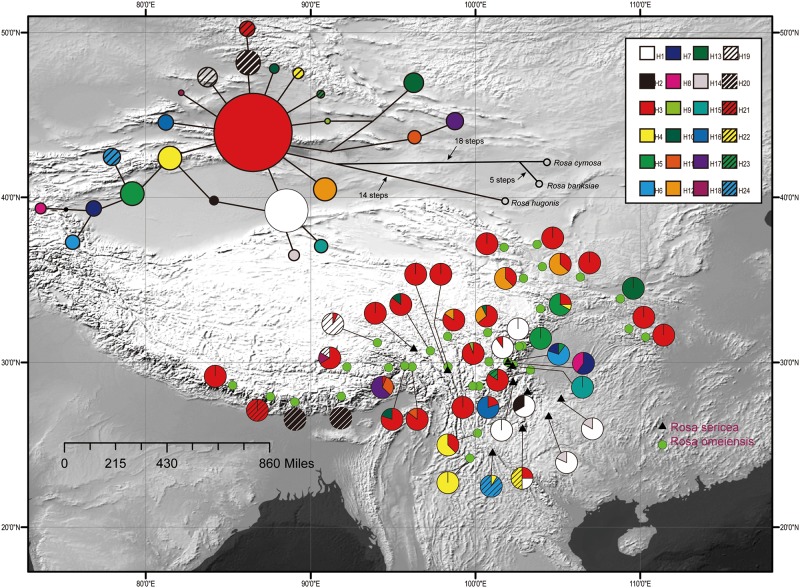
Geographic distribution of 24 cpDNA haplotypes (H1–H24) detected among 42 populations of *R. sericea* and *R. omeiensis*. Network of cpDNA haplotypes with outgroup taxa constructed by TCS 1.21. Size of circles in network are proportional to observed frequencies of haplotypes. Map generated in ESRI ArcGIS 10.0 (Esri, 2010).

From 2010–2011, we collected 459 samples from 42 populations representing *R. sericea* and *R. omeiensis* from across their distributional ranges (hereafter RS and RO; Figure 2 and Supplementary Table S1). There are several other species of *R. sericea* comprising a species complex, but these species are narrowly endemic with only a few specimen records each and, thus, difficult to assess. For each population, we sampled 10 or more individuals except at sites where the species was rare, and our smallest sample comprised seven individuals (Table 1). Each population consisted of either RS or RO, and there were no populations containing both species based on morphology. Our collections consisted of leaves, which we dried in silica gel in the field and then stored at -80°C until used, and one voucher specimen per population, which we deposited in the herbarium at the Chengdu Institute of Biology, Chinese Academy of Sciences (CDBI, Table 1). We also recorded the geolocations of each sampled population.

**Table 1:** Plant material, sources, detected chlorotypes and the genetic diversity indetified in this study.

Population code	Taxon code^∗^	Location	Latitude (N)	Longitude (E)	Elevation (m)	Sample size	Haplotypes (no. of individuals)	Vouchers	*N*	Hd (SD)	π (SD)
XMZ	RO	Xizang, Maizhokunggar	29.71°	92.2°	4274	6	H3 (4), H18 (1), H19 (1)	GAO XF et al., 13601, CDBI	3	0.600(0.215)	0.00022(0.00009)
XCN	RO	Xizang, Cuona	27.93°	91.85°	3840	8	H20 (8)	GAO XF et al., 13932, CDBI	1	0.000	0.00000
XYD	RO	Xizang, Yadong	27.58°	89.03°	3833	12	H20 (12)	GAO XF et al., 14296, CDBI	1	0.000	0.00000
XDJ	RO	Xizang, Dinggyê	27.91°	87.55°	3900	8	H21 (8)	GAO XF et al., 14487, CDBI	1	0.000	0.00000
XJL	RO	Xizang, Gyirong	28.6°	85.26°	3780	11	H3(11)	GAO XF et al., 14806, CDBI	1	0.000	0.00000
XBR	RO	Xizang, Biru	31.18°	94.04°	3960	13	H3 (1), H19 (12)	GAO XF et al., 15366, CDBI	2	0.154(0.126)	0.00005(0.00004)
SEM	RO	Sichuan, Emeishan	29.53°	103.33°	2890	13	H3 (11), H23 (2)	ZHANG Y 216, CDBI	2	0.282(0.142)	0.00009(0.00005)
SXJ1	RO	Sichuan, Xiaojin	30.99°	102.83°	3337	10	H5 (10)	ZHANG Y 420, CDBI	1	0.356(0.159)	0.00024(0.00011)
SXJ2	RO	Sichuan, Baoxing	30.96°	102.64°	2840	15	H1 (15)	ZHANG Y 421, CDBI	1	0.000	0.00000
SKD2	RO	Sichuan, Kangding	30.01°	101.95°	3525	10	H3 (1), H1 (9)	GAO XF et al., 11856, CDBI	2	0.2(0.154)	0.00007(0.00005)
SLD1	RO	Sichuan, Luding	29.85°	102.28°	3000	10	H7 (6), H8 (4)	GAO XF et al., 11949, CDBI	2	0.533(0.095)	0.00018(0.00003)
SYJ2	RO	Sichuan, Yajiang	30.03°	100.82°	4330	18	H3 (17), H9 (1)	GAO XF et al., 12107, CDBI	2	0.111(0.096)	0.00004(0.00003)
XMK1	RO	Xizang, Markam	29.72°	98.32°	4000	10	H3 (10)	GAO XF et al., 12154, CDBI	1	0.000	0.00000
XBM1	RO	Xizang, Bome1	29.71°	96.15°	3085	10	H3 (8), H10 (2)	GAO XF et al., 12294, CDBI	2	0.356(0.159)	0.00012(0.00005)
XBM2	RO	Xizang, Bome2	29.79°	95.7°	3650	7	H3 (6), H11 (1)	GAO XF et al., 12377, CDBI	2	0.286(0.196)	0.00019(0.00013)
XCY	RO	Xizang, Chagya	30.69°	97.26°	3600	7	H3 (6), H10 (1)	GAO XF et al., 12543, CDBI	2	0.286(0.196)	0.0001(0.00007)
XJD	RO	Xizang, Jomda	31.57°	98.31°	3400	13	H3 (11), H12 (2)	GAO XF et al., 12596, CDBI	2	0.282(0.142)	0.00009(0.00005)
SSD	RO	Sichuan, Seda	31.8°	100.75°	3826	14	H3 (9), H12 (4), H13 (1)	GAO XF et al., 12631, CDBI	3	0.538(0.115)	0.00029(0.00012)
SDC1	RO	Sichuan, Daocheng	28.55°	100.21°	3715	16	H3 (16)	GAO XF et al., 13316, CDBI	1	0.000	0.00000
YZD1	RO	Yunnan, Xianggelila	28.55°	99.83°	4322	10	H3 (2), H16 (8)	GAO XF et al., 13407, CDBI	2	0.356(0.159)	0.00024(0.00011)
XLZ1	RO	Xizang, Nyingchi	29.68°	94.73°	3630	16	H3 (1), H11 (5), H17 (10)	GAO XF et al., 13564, CDBI	3	0.542(0.098)	0.00042( 0.00010)
HXS	RO	Hubei, Xingshan	31.53°	110.33°	2180	17	H3 (17)	GAO XF et al., 13074, CDBI	1	0.000	0.00000
SHPL	RO	Shanxi, Pingli	32.02°	109.31°	2057	7	H3 (7)	GAO XF et al., 13160, CDBI	1	0.000	0.00000
YYD	RO	Yunnan, Yongde	24.17°	99.66°	3148	11	H4 (1), H24 (10)	ZHU ZM et al., 37, CDBI	2	0.182(0.144)	0.00006(0.00005)
YDL	RO	Yunnan, Dali	25.7°	100.11°	3200	8	H3 (3), H4 (5)	ZHU ZM et al., 118, CDBI	2	0.536(0.123)	0.00018(0.00004)
SJZ	RO	Sichuan, Jiuzhaigou	33.24°	103.94°	3055	12	H3 (3), H4 (1), H5 (8)	ZHANG Y 416, CDBI	3	0.530(0.136)	0.0003(0.00008)
QDT	RO	Qinghai, Datong	36.98°	101.73°	2500	13	H3 (13)	GAO XF et al., 15470, CDBI	1	0.000	0.00000
GHZ	RO	Gansu, Hezuo	35.08°	102.92°	2840	8	H3 (3), H12 (5)	GAO XF et al., 15519, CDBI	2	0.536(0.123)	0.00018(0.00004)
SHXA	RO	Shanxi, Xian	33.84°	108.8°	1915	12	H13 (12)	GAO XF et al., 13168, CDBI	1	0.485(0.106)	0.00016(0.00004)
GZL	RO	Gansu, Zhuanglang	35.17°	106.36°	2300	12	H3 (12)	GAO XF et al., 13220, CDBI	1	0.000	0.00000
GJT	RO	Gansu, Jingtai	37.14°	103.74°	2820	14	H3 (14)	GAO XF et al., 13238, CDBI	1	0.000	0.00000
GYZ	RO	Gansu, Yuzhong	35.79°	104.06°	2600	11	H3 (4), H12 (7)	GAO XF et al., 13250, CDBI	2	0.509(0.101)	0.00017( 0.00003)
SKD1	RS	Sichuan, Kangding	30.06°	101.96°	3520	10	H5 (1), H6 (7), H7 (2)	GAO XF et al., 11864, CDBI	3	0.511(0.164)	0.00038(0.00012)
SLD2	RS	Sichuan, Luding	29.84°	102.25°	2350	6	H15 (6)	GAO XF et al., 13286, CDBI	1	0.000	0.00000
XMK2	RS	Xizang, Markam	29.57°	98.31°	3287	9	H3 (9)	GAO XF et al., 12183, CDBI	2	0.5(0.128)	0.00084(0.00022)
XLL2	RS	Xizang, Lhorong2	30.88°	96.26°	3880	7	H3 (7)	GAO XF et al., 12513, CDBI	1	0.000	0.00000
YJD	RS	Yunnan, Jingdong	24.55°	101.03°	2454	12	H4 (12)	ZHU ZM et al., 77, CDBI	1	0.000	0.00000
YLQ	RS	Yunnan, Luquan	26.02°	102.85°	2650	8	H1 (2), H3 (2), H22 (4)	ZHU ZM et al., 153, CDBI	3	0.714(0.123)	0.00034(0.00007)
SMG	RS	Sichuan, Meigu	28.25°	103.16°	3150	13	H3 (13)	ZHU ZM et al., 195, CDBI	1	0.000	0.00000
SMN1	RS	Sichuan, Mianning	28.85°	102.28°	2400	9	H1 (6), H2 (3)	GAO XF et al., 11632, CDBI	2	0.500(0.128)	0.00017(0.00004)
YWX	RS	Yunnan, Weixin	27.84°	105.21°	1450	11	H1 (9), H14 (2)	GAO XF et al., 12818, CDBI	2	0.327(0.153)	0.00011(0.00005)
GUWN	RS	Guizhou, Weining	26.76°	104.43°	2300	12	H1 (10), H14 (2)	GAO XF et al., 12858, CDBI	2	0.303(0.147)	0.0001(0.00005)
Total									24	0.776(0.019)	0.00062(0.00003)


*^∗^RO, Rosa omeiensis; RS, Rosa sericea.*

### DNA Extraction, Sequencing, and Microsatellite Genotyping

We extracted total genomic DNA from the dried leaf tissue via a modified cetyltrimethyl ammonium bromide (CTAB) method (Doyle and Doyle, 1987). In a preliminary screening, we surveyed 12 intergenic spacer (IGS) regions of the chloroplast (cp) genome of RS and RO (data not shown). Based on these results, we used the cpDNA regions *tab*E-*ndh*J, *trn*L-*trn*F, and *ndh*F-*rpl*32 for further analysis due to their high levels of polymorphism and amplification efficiency. Additionally, we amplified the second exon of LEAFY, a floral meristem identity gene, initially using primers designed by Ritz et al. (2011) and then with primers designed specifically for this study (RSLEAFY-2F, 5′-GCTGCGGAGGATTAGGAGAGGAGT-3′, RSLEAFY-2R, 5′-GCAGCGCATAGCAGTGAACATAGT-3′). We sampled LEAFY for the ingroup only. We performed all amplifications using standard PCR in 25 μL volume with ca. 50 ng of template DNA, 2.5 μL of 10×PCR buffer, 2 μL of 2.5 mM dNTPs, 0.5 μL of 10 μM each primer and 0.5 unit of TaKaRa ExTaq (Takara Biomedical Technology, Co., Ltd., Beijing, China). The PCR cycle for all three cpDNA fragments was as follows: enzyme activation at 94°C for 3 min, followed by 35 cycles of denaturation at 94°C for 1 min, annealing at 55°C for 1 min and extension at 72°C for 1.5 min; followed by a final extension of 72°C for 10 min. We purified the PCR products using standard methods and sequenced them on an ABI PRISM 3730 (Applied Biosystems) using the same primers for PCR. For the resulting sequences, we performed manual editing in SeqMan Pro 7.1 (implemented in DNASTAR, Lasergene) and sequence alignment in MEGA 4 (Tamura et al., 2007) using the ClustalX method (Thompson et al., 1994, 1997) under default settings. We removed all indels, which were exclusively repeats of adjacent bases and unstable in sequencing. For LEAFY, we extracted independent, polymorphic alleles, or haplotypes, using the algorithm for phasing in DnaSP (Librado and Rozas, 2009) with an output threshold of 0.7 (Stephens et al., 2001; Stephens and Donnelly, 2003). We deposited all cpDNA and LEAFY haplotypes in GenBank under Accession Nos. KF850715–KF850751, KF850756–KF850792, KF851050–KF851086, and MH258749–MH258789. We also genotyped eight EST-nSSR loci in the RS and RO using primers and amplification protocols developed for the *Rosa* genus (see Supplementary Table S2). In a preliminary analysis, we investigated 20 pairs of EST primers, and, based on the results, we selected eight highly polymorphic primer pairs that could produce good quality amplicons (Gao et al., 2015b). We separated the PCR amplicons on a MegaBACE 1000 (GE Healthcare Biosciences, Sunnyvale, CA, United States) and scored alleles manually using GENETIC PROFILER software (version 2.2; GE Healthcare Biosciences).

### Population Genetic and Phylogeographic Data Analyses

To verify congruence in phylogenetic signal among the three cpDNA fragments, we carried out a partition-homogeneity test (Farris et al., 1994) using the software PAUP^∗^ 4.0b (Swofford, 2003). Based on the results, we used the combined cpDNA data set for all subsequent analyses. For the combined cpDNA, we analyzed sequence variation in MEGA4 (Tamura et al., 2007) and calculated nucleotide (*π*) and haplotype (*h*) diversity (Nei, 1987) in DnaSP 5 (Librado and Rozas, 2009). We also compared population differentiation for phylogenetically ordered (*N*_ST_) and unordered (*G*_ST_) haplotypes and for all populations using PERMUT1.0 (Pons and Petit, 1996). We used the results and a test of significance comprising 1000 permutations to determine if *N*_ST_ > *G*_ST_, which constitutes a test for phylogeographic structure. We investigated the phylogenetic relationships among populations (represented by haplotypes) using the statistical parsimony procedure for phylogenetic network estimations implemented in TCS 1.21 (Clement et al., 2000) with a 95% criterion for parsimonious connections. We also reconstructed a phylogeny from all unique chloroplast haplotypes with maximum likelihood (ML) and neighbor-joining (NJ) methods with four accessions of three species, *Rosa hugonis* Hemsl. *R. cymosa* Tratt. and *R. banksiae* R. Br., constituting the outgroup based on the framework of *Rosa* phylogeny (Fougere-Danezan et al., 2015). The accession numbers from GenBank are as follows: *R. hugonis* (two accessions), *R. banksiae* and *R. cymosa* (KF850752–KF850755, KF850793–KF850796, KF851087–KF851090). We conducted the NJ analysis with MEGA7 (Kumar et al., 2016), incorporating the Kimura 2-parameter (K2P) model of DNA evolution. To evaluate support for clades, we performed 100 bootstrap replicates. For the ML estimate, we first determined partitions and models of evolution for each within PartitionFinder v.2.1.1 (Lanfear et al., 2017). We performed the ML analysis in RAxML HPC BlackBox (version 8.2.10) (Stamatakis, 2014) with 100 bootstrap replicates. We conducted NJ and ML analyses and the analysis in PartitionFinder2 on the Cipres Science Gateway (Miller et al., 2010).

Additionally, we constructed a network of the unphased sequences of LEAFY using SplitsTree4 (Huson and Bryant, 2006) and implementing the NeighborNet algorithm with Kimura 2-parameter (K2P) distances and ordinary least squares inference of branch lengths. We performed a bootstrap analysis in SplitTree4 with 1000 replicates and we recorded the bootstrap values for major splits.

To identify population groups and genetic barriers, we conducted a spatial analysis of molecular variance (SAMOVA) in SAMOVA 1.0 (Dupanloup et al., 2002) on plastid makers. The SAMOVA algorithm uses geolocations of populations and genetic data to generate a user-defined number of geographically adjacent groups (K) that have maximal among-group genetic variability and minimal within-group variability. Our SAMOVA analyses comprised five independent analyses of 1000 iterations each for K = 2–20, and the asymptotic shape of the distribution of SAMOVA results showed that this range K was sufficient to capture the best-fit number of groups. We compared the groups inferred using SAMOVA to determine if they exhibited distinguishing morphological characters. We also performed analyses of molecular variance (AMOVA) (Excoffier et al., 1992) in the software package ARLEQUIN 3.5 (Excoffier et al., 2005) using all the populations under the null hypotheses that only one species exists.

For the nSSRs, we calculated the total number of detected alleles (N_A_), allelic richness (R_S_), and gene diversity (H_S_). We also computed differentiation between populations using F_ST_ (Weir and Cockerham, 1984), for which we determined significance at each locus and overall using 1000 bootstrap permutations. We performed all analyses of the nSSR data in GenALEx (version 6.501; Peakall and Smouse, 2012) and GENEPOP version 4.0 (Raymond and Rousset, 1995). We used the complete nSSR dataset (i.e., 42 populations) to infer population structure in STRUCTURE version 2.3 (Pritcharda et al., 2009), which we ran under the admixture model with independent allele frequencies for 100,000 Markov chain Monte Carlo (MCMC) generations following 10,000 burn-in generations. We performed 10 replications each in STRUCTURE for K = 1–20, and we selected the optimal K according to Evanno et al., 2005) for downstream analyses.

### Present Distribution Modeling and ENM Identity Test

We sought to compare and contrast the geographic ranges and environmental tolerances of putative species within RS and RO. Unfortunately, the only preexisting, comprehensive, geographic range maps for these species have coverage limited to China (Fang et al., 2011), and even these may inadvertently integrate ongoing taxonomic controversies and confusion within RS and RO. Therefore, we performed presence-only ecological niche modeling (ENM) in MaxEnt 3.3.3k (Phillips et al., 2006) to infer species environmental tolerances and, consequently, their probable geographic ranges. We generated ENMs using occurrence data from our field collections as well as vetted specimens from A, BM, CDBI, E, HNWP, IBSC, K, KATH, KUN, PE, TUCH, and WUK and vetted specimen records in the Global Biodiversity Information Facility (GBIF) and the Chinese Virtual Herbarium (CVH). For all herbarium records, we verified the taxonomic identity by personally examining the specimens or specimen images, and we ensured that geolocations were consistent with known ranges. In total, we obtained 354 high quality records that were spatially unique at 2.5 arc minutes resolution (∼5 km^2^ at the equator) representing 121 RS and 233 RO. We used 19 temperature, perception, and seasonality variables from WorldClim (Hijmans et al., 2005^1^), also at 2.5 arc minutes resolution, to generate the ENMs in MaxEnt. We assessed model quality with cross-validation comprising 100 replicates using 25% of the data for model testing, and we evaluated the accuracy of each cross validation test using the area under the ROC curve (AUC) (Fawcett, 2006).

### Identity Test and Niche Overlap Test

We compared the environmental niches of the two putative species using niche-identity tests, in which the difference between the actual niches were contrasted with null models generated from randomly reshuffled occurrence points (Warren et al., 2008). We calculated niche identity using Schoener’s *D* similarity index (Schoener, 1968) and Warren’s *I* (Warren et al., 2008) implemented in ENMTools (Warren et al., 2010). We conducted 100 pseudoreplicates of shuffling to generate null models for these statistics.

### Principal Component Analysis (PCA) of Environment Factors

We used principal components analyses (PCA) to better understand and visualize the similarities and differences between the environments in which RS and RO occur. We conducted the PCAs using the same 19 temperature, precipitation, and seasonality variables used for ENMs, as well as average high and low temperatures and precipitation for each of 12 months from WorldClim (Hijmans et al., 2005; Fick and Hijmans, 2017) and elevation, which we obtained from specimen records. For each variable, we extracted values in ArcGIS (Esri, 2010) for the same 354 occurrence data points used for ENM, and we performed the PCAs on matrices of the environmental values standardized using *z*-scores. We simultaneously visualized the distributions of species in environmental space and along a potential elevational gradient by adding elevation contours to the PCA plots using the Vegan library (Dixon, 2003). We also visualized the same data according to groups of populations identified using STRUCTURE. Additionally, we performed a more stringent PCA analysis using only the data points comprising our field collections, which are the best vetted for geolocation and taxonomic identity. Within this more stringent analysis, we reduced the number of variables to one each from among UPGMA clusters in order to limit redundancy due to co-variance. We performed the UPGMA in R using a core hierarchical clustering function on the standardized environmental variables, and we selected variables from clusters more divergent than height five, where height is roughly equivalent to units of standard deviation.

## Results

### Sequence Characteristics and Within-Population Genetic Diversity

The aligned cpDNA-IGS data represented all 459 individuals from 42 populations and comprised 3057 bp (2962 bp when indels were excluded) with 26 single nucleotide polymorphisms, of which 25 were parsimony informative. There were also 10 homopolymeric indels ranging from 1 to 24 bp that we removed. From among the 459 individuals, we identified 24 unique haplotypes based on single nucleotide polymorphisms. Among the haplotypes, H3 exhibits a broad east-west distribution across the northern range of RS and RO within the southwestern mountains of China and adjacent areas, and H1 is regionally common within the southeastern part of the range (i.e., southeastern Hengduan Mountains, Figure 2). The network analysis shows haplotype H3 in a central position with many connections, suggesting that it is ancestral to the others (Figure 2). However, chloroplast data resolved no significant differences between these two putative species.

The matrix for phylogeny represented 25 haplotypes and 4 samples comprising the outgroup, and consisted of 3068 bp with 60 variable sites, of which 38 were parsimony informative. PartitionFinder yielded two partitons (*trn*L-*trn*f + *tab*E-*ndh*J, *ndh*F-*rpl*32) with the best models as GTR and GTR + I, respectively. Topologies generated by NJ and ML were congruent in terms of major clades, thus only the NJ tree is presented in Supplementary Figure S1. The monophyly of RS-RO was strongly supported with NJ bootstrap values (87%), although lineages within RS-RO were not well-resolved.

Genetic statistics representing the cpDNA generally revealed high diversity values. In particular, genetic differentiation (*G*_ST_ = 0.695, *N*_ST_ = 0.811) was high, and *N*_ST_ was greater than *G*_ST_ for all populations and in the metapopulation (*p* < 0.01, Supplementary Table S1) based on permutation tests, suggesting the existence of phylogeographic structure. Moreover, the total nucleotide (*π*) and haplotype (*H*_d_) diversity across the metapopulation were 0.00062 and 0.766, respectively, and genetic diversity for the metapopulation (*H*_T_ = 0.790) was higher than the average diversity within populations (*H*_S_ = 0.241).

**FIGURE 3:**
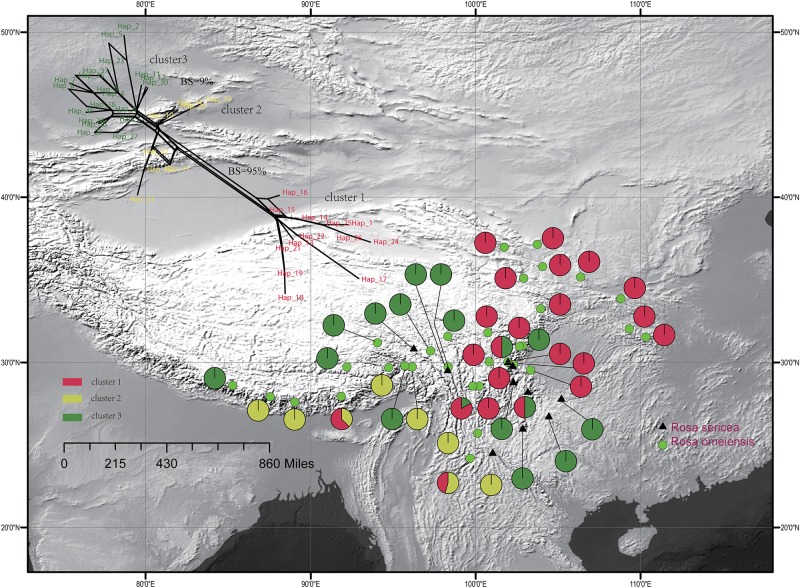
Geographic distribution of the 42 populations of the *R. sericea* complex color-coded according to the network analysis of the phased genetic sequences from LEAFY. Network shown at top and constructed in SplitsTree. Map generated in ESRI ArcGIS 10.0 (Esri, 2010).

The single copy nuclear gene, LEAFY, comprised 752 bi-parental alleles of 619 bp in alignment with 38 variable sites representing 41 unique haplotypes. The haplotypes were clustered in three groups within the network analysis, which showed moderate support a split between cluster I and clusters II and III (95% bootstrap support (BS), 500 replicates, Figure 3), and lower support for a split between cluster III and the others (<10% BS; Figure 3). Based on the LEAFY haplotypes, we found that total nucleotide (*π*) and haplotype (*H*_d_) diversity in all populations was 0.010 and 0.937. Genetic diversity calculated for all populations (*H*_T_ = 0.950) was higher than the average intrapopulation diversity (*H*_S_ = 0.520). We also detected significant phylogeographic structure due to genetic differentiation based on *N*_ST_ (0.798) being much greater than *G*_ST_ (0.452) in all populations and within the metapopulation (*p* < 0.01, Supplementary Table S1).

### Nuclear Microsatellite Genotyping

From the 42 populations representing the RS-RO, we detected a total of 186 alleles from among the eight nSSR loci surveyed, and we inferred high mean per-locus allele and gene diversity (N_A_ = 23.25; H_S_ = 0.578; H_T_ = 0.806; see Supplementary Table S2). Population differentiation (*F_ST_*) was significant at each locus (*P* < 0.05) and averaged across all loci (0.283; Supplementary Table S2).

**FIGURE 4:**
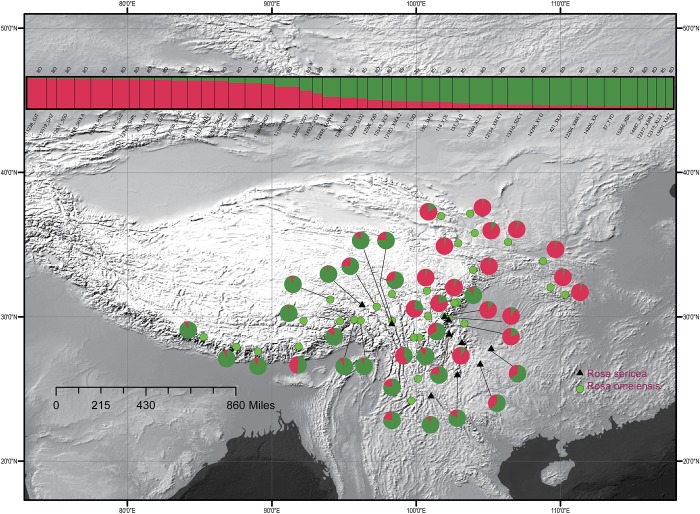
Geographic distribution of the 42 populations of the *R. sericea* complex color-coded according their groups inferred with analysis in STRUCTURE of eight nuclear microsatellite (nSSR) loci. Histogram of the STRUCTURE assignment test for 42 populations (507 individuals) shown at top. Population codes and abbreviation for species are identified in Table 1. Map generated in ESRI ArcGIS 10.0 (Esri, 2010).

For the entire nSSR dataset (42 populations, *n* = 507), STRUCTURE yielded the highest likelihood when samples were clustered into two groups (K = 2, Supplementary Figure S2). The STRUCTURE results showed distinct geographic patterns (Figure 4); namely western, northwestern, and northeastern populations were more genetically isolated than central and south-central populations, which were more admixed (Figure 4). However, there was no observable relationship between the clusters inferred in STRUCTURE and recognized species within RS and RO or between the clusters and morphology. The two major clusters revealed by nSSR were largely congruent with the two highly supported clusters in LEAFY (Figure 3, 4; excluding the weakly supported split between Clusters II and III in LEAFY).

### Population Genetic and Phylogeographic Structure

Spatial genetic analyses of cpDNA haplotypes from 42 populations using SAMOVA indicated that *F*_CT_ increased asymptotically with K clusters from 2 to 20 such that K > 12 did relatively little to improve the clusters by further reducing variation within them (data not shown). None of the 2–20 ways of clustering the populations showed a relationship to morphology or, consequently, to current taxonomic treatments. Therefore, we used taxonomy to divide the sampled populations into two groups for AMOVA analysis (Figure 2 and Table 1) and conducted the analyses on both nuclear and plastid datasets. The AMOVA analyses based on LEAFY (PV = 5.05%, *F*_CT_ = 0.05046, *P* < 0.005, Supplementary Table S3) and cpDNA (PV = 11.52%, *F*_CT_ = 0.11518, *P* < 0.01, Supplementary Table S4) showed a very low level of variation among groups defined according to morphology and classical taxonomy. Thus, neither genetic dataset supports treating RS and RO as independent species. Within species, the LEAFY dataset revealed higher variation within populations rather than among them, while the opposite was true for the cpDNA (Supplementary Table S3).

### Environment Niche Modeling (ENM)

**FIGURE 5:**
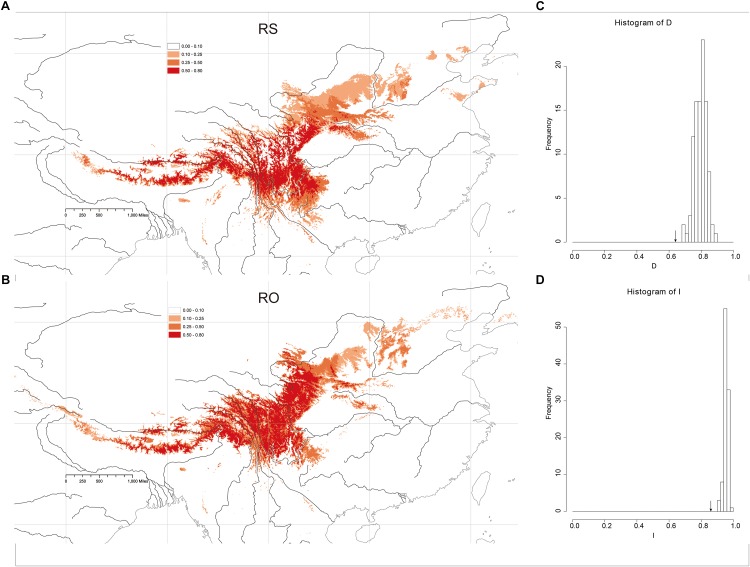
Heat map representing probability of geographic distribution of **(A)** *R. sericea* and **(B)** *R. omeiensis* based on ENM (logistical values from MaxEnt). Shown at 2.5 arc minute resolution and projected based on present day climatic conditions. Occurrence records plotted as black points on maps generated by ESRI ArcGIS 9.3 (Esri, 2010). **(C,D)** Shows histograms of niche identity tests: Schoener’s D and Warren’s I, respectively.

The Maxent models had strong predictive power based on 10 replicates: AUC = 0.989 ± 0.005 (mean ± SD) in RS and AUC = 0.987 ± 0.002, in RO. The models yielded predictions that were similar to the known geographic distributions of both species at a threshold of 0.50 (Figure 5A,B). The main geographic distributional areas for both species were predicted within southwestern China, mainly at high elevations. Though the modeled ranges of the species overlap, the range of RO appears to include higher elevation areas compared to RS.

### Niche Comparation and Principal Component Analysis (PCA)

Niche-identity tests comparing the niches of the two *Rosa* species yielded similarity values of 0.860 according to Warren’s *I* and 0.609 based on Schoener’s D. Comparisons of the observed *I* and *D* values to null distributions showed that the species did not occupy identical niches (*P* < 0.01, *P* < 0.01, respectively) (Figure 5C,D). Thus, the niche of each species includes some distinct environments.

**FIGURE 6:**
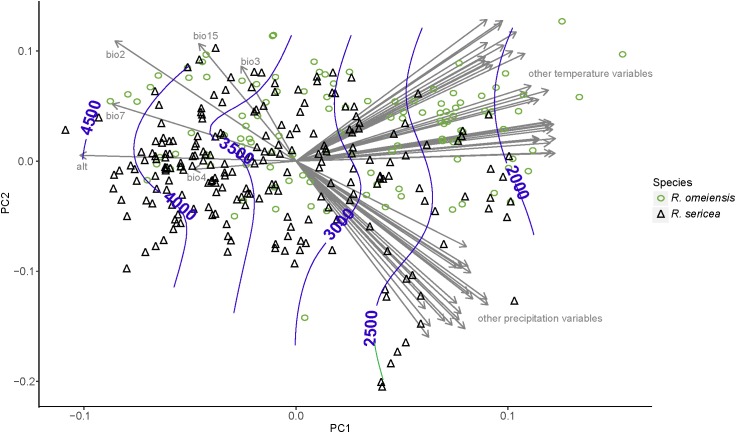
Results of a principal component analysis (PCA) using environmental variables from all vetted occurrence data points, including field collections, herbarium specimens, and records from databases. The variables with the highest loadings on PC axis 1 were: mean diurnal temperature range (bio2), temperature isothermality (bio3), temperature seasonality (bio4), elevation (alt; altitude), temperature annual range (bio7), and precipitation seasonality (bio15). All abbreviations of variables follow WorldClim (Hijmans et al., 2005); http://www.worldclim.org/.

The PCA analyses also do not show strong niche differentiation between RS and RO according to 55 total temperature and precipitation variables (Figure 6). However, some niche differentiation is apparent from PC axis 1. The hierarchical clustering of environmental variables revealed eight distinct clusters of variables, which are also apparent from the plot of our PCA (Figure 6). Among the clusters, the variables with the highest loadings on PC axis 1 were: average precipitation during November (prec11), average minimum temperature during November (temp11), mean diurnal temperature range (bio2), temperature isothermality (bio3), temperature seasonality (bio4), elevation (alt; altitude), temperature annual range (bio7), and precipitation seasonality (bio15). In the analysis using only these variables and our field collected data, clear, though limited, separation of putative species along PC axis 1 was apparent (Supplementary Figure S3). The importance of elevation was apparent from the contours, which showed that the lowest elevation populations were those of RS while the highest elevations were most often RO (Figure 6 and Supplementary Figure S3).

## Discussion

### RS, RO, and Modes and Mechanisms of Divergence

Populations within RS and RO exhibit clear morphological variation (Figure 1), leading to the recognition of several different species, including RS and RO. The morphological differences among entities within RS-RO may be explained by one or several historical or ongoing evolutionary processes. One possible explanation is that RS-RO represents a single, hyperdiverse species. As a hyperdiverse species, RS-RO would likely exhibit genetic panmixia or high levels of gene flow due to more-or-less continuous contact among populations (Hamrick and Godt, 1990; Constance and William, 1991). The morphology of a hyperdiverse species may be intergrading or show some association with the local environment. RS-RO as a single hyperdiverse species comprises a null taxonomic hypothesis (Link-Pérez, 2010) while the alternative is that speciation has occurred or is occurring. If speciation has occurred or is occurring, we would expect to detect clear genetic differentiation, which may be stronger if speciation is complete, or weaker if it is ongoing or confounded by introgression (Nosil, 2008; Liu et al., 2018).

Genetic differentiation between RS and RO is apparent but limited. For example, the AMOVA analyses of the *LEAFY* (Supplementary Table S3) and cpDNA data (Supplementary Table S4) show low to moderate genetic differentiation, respectively (following criteria in Hartl and Clark, 1997). Moreover, the cpDNA haplotypes, notwithstanding haplotype H3, are largely unique within each traditionally recognized taxon even when populations of RS and RO are geographically proximal (Figure 2). Based on these findings, we reject the null hypothesis that the RS-RO comprises one species but suggest that the signal supporting a speciation hypothesis is limited due to confounding effects. The weak signal most likely arises from recent initiation of speciation within the complex and continued gene flow among differentiating lineages (Arnold, 2016).

Speciation in RS-RO may be allopatric, sympatric, or parapatric (Ridley, 2004). Allopatric speciation should be detectable according to separation in geographic space while, sympatric species should co-occur in geographic space. In contrast to these, parapatry should lead to differentiation along an environmental gradient or possibly at the edges of the geographic range (Helbig et al., 2002).

In RS-RO, parapatric speciation, or ecological differentiation, is congruent with our observations in the field and analyses based on ENM (Figure 5) and PCA results (Figure 6 and Supplementary Figure S3). In the field, we observed that several populations of RS-RO represented co-occurrences of the two species along mountainous slopes at lower and higher elevations, respectively (e.g., SLD1 and SLD2, Table 1). The ENM and PCA show incompletely overlapping distributions in geographic and environmental space, respectively, and indicate that the non-overlapping portions of the ranges are at elevational extremes. Therefore, the ENM and PCAs support our observations in the field, which led us to believe that RS-RO is diversifying along an elevational gradient.

Although we suspect that speciation within the RS-RO is best classified as parapatric, we detected isolation by distance in both the cpDNA and LEAFY datasets as would be expected in the case of allopatric speciation. However, isolation by distance is not uncommon even among single, hyperdiverse species (e.g., Andrew et al., 2012). Moreover, a previous study (Gao et al., 2015b) showed recent differentiation between RS and RO, and this helps to rule out allopatry with secondary contact as the explanation for limited observed genetic diversity.

Diversification in RS-RO may have begun during Last Glacial Maximum (LGM, 1.8k yr) (Gao et al., 2015b). During the LGM, the colder climate probably resulted in a lower tree line (Hewitt, 1996, 1999, 2000), generating additional open, alpine habitat suitable for these roses. These alpine habitats may have been broader and more highly connected geographically so that gene flow among populations of RS-RO could readily occur. Within the broadly available alpine habitats, populations constituting RS and RO may have begun to diversify ecologically. Recent initiation of speciation, such as following the LGM, is consistent with the short branches connecting diverse haplotypes of the ancestral type, H3 (Figure 2).

Many prior studies have inferred the importance of Pleistocene glaciations and climatic oscillations (e.g., Sage and Selander, 1975; Rainey and Travisano, 1998) on plant demography in mountainous southwestern China (reviewed in Qiu et al., 2011). During glacial periods, cool-adapted plant species, such as *Rosa*, may have radiated within lower elevations but have had fragmented refugial ranges at higher elevations during interglacials (DeChaine and Martin, 2004; Jaramillo-Correa et al., 2006, 2008; Yuan et al., 2006). Repeated radiation and fragmentation events probably may have facilitated allopatric divergence and opportunities for secondary contact. However, the predominate demographic pattern that we detected in the RS-RO is inconsistent with this commonly inferred scenario, under which we would expect to detect genetically diverse, geographically limited refugial areas with ancestral haplotypes as well as ecological stasis among populations (Constance and William, 1991; Hewitt, 1996, 1999, 2000; Provan and Bennett, 2008; Falcon-Lang and Dimichele, 2010). In contrast, the RS-RO exhibits a widespread ancestral haplotype, H3, broad diversity of genotypes geographically, and ecological divergence, especially for tolerances of elevation range, climatic seasonality, and weather during some of the coldest months of the year (Figure 6 and Supplementary Figure S3).

### Differing Demographic Patterns According to cpDNA and Nuclear DNA

We observed different demographic patterns of isolation by distance based on the cpDNA and nuclear datasets. In particular, the cpDNA data showed notable divergence across a north to south axis (i.e., widespread haplotypes H3 in the north and H1 in the south; Figure 2) while the nuclear data revealed predominant divergence along a northeast–southwest axis (Figure 3, 4). These discordant demographic patterns may result from different dispersal vectors for seeds, which transmit the cpDNA genome, and pollen, which carries one half of the information transmitted within the biparentally-inherited nuclear genome.

Pollen and seed dispersal are known to be important for determining the spatial patterns of gene flow among plant populations, and, for plant species relying on zoonotic pollinators and frugivores as dispersal vectors, animal behaviors are also determinants of demography (Ennos, 1994; Garcia et al., 2007; Krauss et al., 2009; Marsico et al., 2009; Jordano, 2010; Calvino-Cancela et al., 2012). Pollen dispersal in the genus *Rosa* is reportedly carried out by insects, especially Aculeate of Hymenoptera (bees and wasps), Diptera (true flies), and Lepidoptera (moths and butterflies) (summarized in Jacobs et al., 2009), while the seeds of *Rosa* are dispersed within nutritive, fleshy fruits primarily by birds and other vertebrates (Herrera, 1984). Therefore, differences among the behaviors of these animal groups could lead to discordance between the demographies of the cpDNA and nuclear genotypes. Conversely, broad groups of pollinators are largely constrained to plant community types, which are more similar east to west and differ markedly differ more markedly along a steep north-south latitudinal gradient (Stibig et al., 2004; Kato et al., 2008). While pollen dispersal by insects tends to be local and is regarded as “long distance” when it occurs over 100s of meters or a few kilometers (Nason et al., 1996; Schulke and Waser, 2001), pollination across local and “long” distances would be sufficient to maintain gene flow between adjacent populations.

### Species Concept: Integrating Ecology

RS and RO exhibit clear morphological differentiation (e.g., Ku and Robertson, 2003), and in our field studies, we did not observe intergrading morphology, even when populations were geographically proximal or intermixed, except uncommonly among a few individuals. Nevertheless, morphology alone has been insufficiently convincing to resolve taxonomic difficulties within the RS-RO because these traits could represent acclimation instead of evolutionary adaptation. This is especially true given limited genetic variability between the putative species and the occasional individual with intermediate traits. Thus, only by integrating ecology, are we able to infer recent or ongoing speciation within RS-RO and show support for the existing taxonomic opinion that *R. sericea* and *R. omeiensis* represent unique, divergent or diverging entities. Ecological features are now widely used as a component of species delimitation as well as to detect cryptic species [e.g., Madagascar day geckos and other vertebrate species in Madagascar (Raxworthy et al., 2003, 2007)].

The traits that distinguish RS and RO are likely to have ecological importance as adaptations along the environmental gradient over which these two species are diverging. In RS, the smaller number of leaflets may represent an adaptation to temperature, which is higher in the lower elevations where the species occurs and, thus, increases potential for water loss. Plants can mitigate water loss in higher temperature environments with smaller leaf surface areas (Givnish and Kriebel, 2017), such as from the smaller number of leaflets in RS. The relationship of pedicel thickness to the environments favored by RS and RO is more difficult to assess. However, in general, greater investment in fruits is known to be correlated with attracting dispersal vectors and providing protection and nutrition for embryos (Salisbury, 1942).

Several recent proposals for species concepts assert the importance of integration of ecology with morphology and genetic evidence. For example, under the unified species concept of De Queiroz (2005, 2007), ecological divergence is one of many equally important lines of evidence that can support the existence of a unique evolutionary lineage. Similarly, Freudenstein et al. (2017), argued that species are distinguished within lineages by having unique ecological roles, which represent the interactions of their morphology with the environment. Although these modern concepts have highlighted the importance of integrating ecology alongside other aspects of species biology into taxonomic thinking, the notion that speciation is influenced or mitigated by ecological interactions dates at least to the 1940s (Mayr, 1942; Schluter, 2001) if not to Darwin and Wallace (1858), who posited that environmental factors were the mechanisms of natural selection. Here, we loosely adopt the highly theoretical unified species concept of De Queiroz, 2007) for RS and RO, between which we show evidence of ecological divergence and in which we and others have observed clear morphological differences. Future studies may examine the ecological impacts of the morphological differences between the two species and develop a more mechanistic taxonomic concept consistent with Freudenstein et al. (2017).

## Conclusion

While a traditional genetics method failed to recover a clear boundary between two morphologically distinct, well-recognized entities, *R. sericea* and *R. omeiensis*, we detected differing ecological preferences along an environmental gradient between these entities suggesting ongoing parapatric speciation. We advocate that the clear morphological and ecological differences *R. sericea* and *R. omeiensis* are sufficient for their delimitation as independent species. In fact, species concepts require integrated evidence such as monophyly, genetic diversity, ecology, morphology, and so on to delimit evolutionarily unique entities, because evolution is continuous and leaves different, detectable footprints within different groups of organisms. Moreover, integrative species concepts are particularly important for plants, which sometimes maintain gene flow at low levels even among long-diverged lineages. Recognition of unique entities, such as *R. sericea* and *R. omeiensis*, has important implications for conservation decisions and robust estimates of biodiversity.

## Author Contributions

X-FG: conceive and design the experiments. Y-DG: perform the experiments. Y-DG and AH: analysis the data. X-FG, Y-DG, and AH: contributed reagents, materials, analysis tools, and wrote the manuscript.

## Conflict of Interest Statement

The authors declare that the research was conducted in the absence of any commercial or financial relationships that could be construed as a potential conflict of interest.

## Footnotes

1 http://www.worldclim.org/

## References

[B1] Abbott R. J. Brennan A. C. (2014). Altitudinal gradients, plant hybrid zones and evolutionary novelty. *Philos. Trans. R. Soc. B Biol. Sci.* 369:20130346. 10.1098/rstb.2013.0346 24958920 PMC4071520

[B2] Agapow P. M. Bininda-Emonds O. R. Crandall K. A. Gittleman J. L. Mace G. M. Marshall J. C. (2004). The impact of species concept on biodiversity studies. *Q. Rev. Biol.* 79 161–179. 10.1086/383542 15232950

[B3] Aldhebiani A. Y. (2018). Species concept and speciation. *Saudi J. Biol. Sci.* 25 437–440. 10.1016/j.sjbs.2017.04.013 29686507 PMC5910646

[B4] An Z.-S. Kutzbach J. E. Prell W. L. Porter S. C. (2001). Evolution of Asian monsoons and phased uplift of the himalaya-tibetan plateau since late miocene times. *Nature* 411 62–66. 10.1038/35075035 11333976

[B5] Andrew R. L. Ostevik K. L. Ebert D. P. Rieseberg L. H. (2012). Adaptation with gene flow across the landscape in a dune sunflower. *Mol. Ecol.* 21 2078–2091. 10.1111/j.1365-294X.2012.05454.x 22429200

[B6] Arnold M. L. (2016). *Divergence With Genetic Exchange.* Oxford: Oxford University Press.

[B7] Arrhenius O. (1921). Species and area. *J. Ecol.* 9 95–99. 10.2307/2255763

[B8] Bond W. J. (1989). The tortoise and the hare: ecology of angiosperm dominance and gymnosperm persistence. *Biol. J. Linnean Soc.* 36 227–249. 10.1111/j.1095-8312.1989.tb00492.x

[B9] Calvino-Cancela M. Escudero M. Rodriguez-Perez J. Cano E. Vargas P. Velo-Anton G. (2012). The role of seed dispersal, pollination and historical effects on genetic patterns of an insular plant that has lost its only seed disperser. *J. Biogeogr.* 39 1996–2006. 10.1111/j.1365-2699.2012.02732.x

[B10] Caro L. Caycedo-Rosales P. Bowie R. Slabbekoorn H. Cadena C. (2013). Ecological speciation along an elevational gradient in a tropical passerine bird? *J. Evol. Biol.* 26 357–374. 10.1111/jeb.12055 23298144

[B11] Carpenter C. (2005). The environmental control of plant species density on a Himalayan elevation gradient. *J. Biogeogr.* 32 999–1018. 10.1111/j.1365-2699.2005.01249.x

[B12] Ceballos G. Ehrlich P. R. Barnosky A. D. García A. Pringle R. M. Palmer T. M. (2015). Accelerated modern human–induced species losses: entering the sixth mass extinction. *Sci. Adv.* 1:e1400253. 10.1126/sciadv.1400253 26601195 PMC4640606

[B13] Chen F. B. (1992). H-D event: an important tectonic event of the late Cenozoic in Eastern Asia. *Mt. Res.* 10:8.

[B14] Chen F. B. (1996). Second discussion on the H-D movement. *Mt. Res.* 17:9.

[B15] Clement M. Posada D. Crandall K. A. (2000). TCS: a computer program to estimate gene genealogies. *Mol. Ecol.* 9 1657–1659. 10.1046/j.1365-294x.2000.01020.x 11050560

[B16] Colin E. H. Guy W. A. (2015). The ubiquity of alpine plant radiations: from the andes to the Hengduan Mountains. *New Phytol.* 207 275–282. 10.1111/nph.13230 25605002

[B17] Constance I. M. William J. L. (1991). “Strategies for conserving clinal, ecotypic, and disjunct population diversity in widespread species,” in *Genetics and Conservation of Rare Plants*, ed. Falk D. A. (Oxford: Oxford University Press), 283.

[B18] Coyne J. A. Orr H. A. (2004). *Speciation.* Sunderland, MA: Sinauer.

[B19] Darwin C. Wallace A. R. (1858). On the tendency of species to form varieties; and on the perpetuation of varieties and species by natural means of selection. *Zool. J. Linnean Soc.* 3 45–62. 10.1111/j.1096-3642.1858.tb02500.x

[B20] De Queiroz K. (2005). Different species problems and their resolution. *Bioessays* 27 1263–1269. 10.1002/bies.20325 16299765

[B21] De Queiroz K. (2007). Species concepts and species delimitation. *Syst. Biol.* 56 879–886. 10.1080/10635150701701083 18027281

[B22] DeChaine E. G. Martin A. P. (2004). Historic cycles of fragmentation and expansion in *Parnassius smintheus* (papilionidae) inferred using mitochondrial DNA. *Evolution* 58 113–127. 10.1111/j.0014-3820.2004.tb01578.x 15058724

[B23] Dixon P. (2003). VEGAN, a package of R functions for community ecology. *J. Veg. Sci.* 14 927–930. 10.1111/j.1654-1103.2003.tb02228.x

[B24] Doyle J. J. Doyle J. L. (1987). A rapid DNA isolation procedure for small quantities of fresh leaf tissue. *Phytochem. Bull.* 19 11–15.

[B25] Dupanloup I. Schneider S. Excoffier L. (2002). A simulated annealing approach to define the genetic structure of populations. *Mol. Ecol.* 11 2571–2581. 10.1046/j.1365-294X.2002.01650.x 12453240

[B26] Ennos R. A. (1994). Estimating the relative rates of pollen and seed migration among plant-populations. *Heredity* 72 250–259. 10.1038/hdy.1994.35

[B27] Esri. (2010). *ArcGIS. Version v.10*. Redlands, CA: ESRI.

[B28] Evanno G. Regnaut S. Goudet J. (2005). Detecting the number of clusters of individuals using the software STRUCTURE: a simulation study. *Mol. Ecol.* 14 2611–2620. 10.1111/j.1365-294X.2005.02553.x 15969739

[B29] Excoffier L. Laval G. Schneider S. (2005). ARLEQUIN (version 3.0), an integrated software package for population genetic data analysis. *Evol. Bioinform. Online* 1 47–50. 10.1177/117693430500100003 19325852 PMC2658868

[B30] Excoffier L. Smouse P. E. Quattro J. M. (1992). Analysis of molecular variance inferred from metric distances among DNA haplotypes, application to human mitochondrial DNA restriction data. *Genetics* 131 479–491. 1644282 10.1093/genetics/131.2.479 PMC1205020

[B31] Falcon-Lang H. J. Dimichele W. A. (2010). What happened to the coal forests during Pennsylvanian glacial phases? *PALAIOS* 25 611–617. 10.2110/palo.2009.p09-162r

[B32] Fang J. Y. Wang Z. H. Wang Z. Y. (2011). *Atlas of Woody Plants in China: Distribution and Climate.* Berlin: Springer Science & Business Media 10.1007/978-3-642-15017-3

[B33] Farris J. S. Kallersjo M. Kluge A. G. Bult C. (1994). Testing significance of incongruence. *Cladistics* 10 315–319. 10.1111/j.1096-0031.1994.tb00181.x

[B34] Fawcett T. (2006). An introduction to ROC analysis. *Pattern Recognit. Lett.* 27 861–874. 10.1016/j.patrec.2005.10.010

[B35] Fick S. E. Hijmans R. J. (2017). WorldClim 2: new 1-km spatial resolution climate surfaces for global land areas. *Int. J. Climatol.* 37 4302–4315. 10.1038/sdata.2018.254 30422125 PMC6233254

[B36] Florio A. M. Ingram C. M. Rakotondravony H. A. Louis E. E. Raxworthy C. J. (2012). Detecting cryptic speciation in the widespread and morphologically conservative carpet chameleon (*Furcifer lateralis*) of Madagascar. *J. Evol. Biol.* 25 1399–1414. 10.1111/j.1420-9101.2012.02528.x 22686488

[B37] Fougere-Danezan M. Joly S. Bruneau A. Gao X. F. Zhang L. B. (2015). Phylogeny and biogeography of wild roses with specific attention to polyploids. *Ann. Bot.* 115 275–291. 10.1093/aob/mcu245 25550144 PMC4551085

[B38] Freudenstein J. V. Broe M. B. Folk R. A. Sinn B. T. (2017). Biodiversity and the species concept lineages are not enough. *Syst. Biol.* 66 644–656. 10.1093/sysbio/syw098 27798406

[B39] Funk D. J. Omland K. E. (2003). Species-level paraphyly and polyphyly: frequency, causes, and consequences, with insights from animal mitochondrial DNA. *Annu. Rev. Ecol. Evol. Syst.* 34 397–423. 10.1016/j.ympev.2009.08.024 19716428

[B40] Gao Y. D. Harris A. J. He X. J. (2015a). Morphological and ecological divergence of Lilium and Nomocharis within the Hengduan Mountains and Qinghai-Tibetan plateau may result from habitat specialization and hybridization. *BMC Evol. Biol.* 15:147. 10.1186/s12862-015-0405-2 26219287 PMC4518642

[B41] Gao Y. D. Zhang Y. Gao X. F. Zhu Z. M. (2015b). Pleistocene glaciations, demographic expansion and subsequent isolation promoted morphological heterogeneity: a phylogeographic study of the alpine rosa sericea complex (Rosaceae). *Sci. Rep.* 5:11698. 10.1038/srep11698 26123942 PMC5155592

[B42] Gao Y. D. Harris A. J. Zhou S. D. He X. J. (2013). Evolutionary events in Lilium (including Nomocharis, Liliaceae) are temporally correlated with orogenies of the Q-T plateau and the Hengduan Mountains. *Mol. Phylogenet. Evol.* 68 443–460. 10.1016/j.ympev.2013.04.026 23665039

[B43] Garcia C. Jordano P. Godoy J. A. (2007). Contemporary pollen and seed dispersal in a Prunus mahaleb population: patterns in distance and direction. *Mol. Ecol.* 16 1947–1955. 10.1111/j.1365-294X.2006.03126.x 17444903

[B44] Gavrilets S. Li H. Vose M. D. (2000). Patterns of parapatric speciation. *Evolution* 54 1126–1134. 10.1111/j.0014-3820.2000.tb00548.x 11005282

[B45] George G. S. (1951). The species concept. *Evolution* 5 285–298. 10.1111/j.1558-5646.1951.tb02788.x

[B46] Givnish T. J. Kriebel R. (2017). Causes of ecological gradients in leaf margin entirety: evaluating the roles of biomechanics, hydraulics, vein geometry, and bud packing. *Am. J. Bot.* 104 354–366. 10.3732/ajb.1600287 28232316

[B47] Givnish T. J. Millam K. C. Mast A. R. Paterson T. B. Theim T. J. Hipp A. L. (2009). Origin, adaptive radiation and diversification of the Hawaiian lobeliads (Asterales: Campanulaceae). *Proc. R. Soc. B Biol. Sci.* 276 407–416. 10.1098/rspb.2008.1204 18854299 PMC2664350

[B48] Guo Q. Kelt D. A. Sun Z. Liu H. Hu L. Ren H. (2013). Global variation in elevational diversity patterns. *Sci. Rep.* 3:3007. 10.1038/srep03007 24157658 PMC6505670

[B49] Hamrick J. L. Godt M. W. (1990). “Allozyme diversity in plant species,” in *Plant Population Genetics, Breeding, and Genetic Resources*, eds Brown A. H. D. Clegg M. T. Kahler A. L. Weir B. S. (Sunderland, MA: Sinauer), 43–63.

[B50] Hardin J. W. (1957). Studies in the Hippocastanaceae, IV. hybridization in aesculus. *Rhodora* 59 185–203.

[B51] Harris A. J. Xiang Q.-Y. Thomas D. T. (2009). Phylogeny, origin, and biogeographic history of *Aesculus L*. (Sapindales) an update from combined analysis of DNA sequences, morphology, and fossils. *Taxon* 58 108–126. 10.1002/tax.581012

[B52] Harrison R. G. (2012). The language of speciation. *Evolution* 66 3643–3657. 10.1111/j.1558-5646.2012.01785.x 23206125

[B53] Harrison R. G. Larson E. L. (2014). Hybridization, introgression, and the nature of species boundaries. *J. Heredity* 105(Suppl. 1), 795–809. 10.1093/jhered/esu033 25149255

[B54] Hartl D. L. Clark A. G. (1997). *Principles of Population Genetics.* Sunderland, MA: Sinauer Associates.

[B55] Helbig A. J. Knox A. G. Parkin D. T. Sangster G. Collinson M. (2002). Guidelines for assigning species rank. *IBIS* 144 518–525. 10.1046/j.1474-919X.2002.00091.x

[B56] Herrera C. M. (1984). Seed dispersal and fitness determinants in wild rose: combined effects of hawthorn, birds, mice, and browsing ungulates. *Oecologia* 63 386–393. 10.1007/BF00390670 28311216

[B57] Hewitt G. M. (1996). Some genetic consequences of ice ages, and their role in divergence and speciation. *Biol. J. Linnean Soc.* 58 247–276. 10.1111/j.1095-8312.1996.tb01434.x 15101575

[B58] Hewitt G. M. (1999). Post-glacial re-colonization of European Biota. *Biol. J. Linnean Soc.* 68 87–112. 10.1111/j.1095-8312.1999.tb01160.x

[B59] Hewitt G. M. (2000). The genetic legacy of the Quaternary ice ages. *Nature* 405 907–913. 10.1038/35016000 10879524

[B60] Hey J. (2006). On the failure of modern species concepts. *Trends Ecol. Evol.* 21 447–450. 10.1016/j.tree.2006.05.011 16762447

[B61] Hijmans R. Cameron S. Parra J. Jones P. Jarvis A. (2005). Very high resolution interpolated climate surfaces for global land areas. *Int. J. Climatol.* 25 1965–1978. 10.1038/sdata.2018.254 30422125 PMC6233254

[B62] Huson D. H. Bryant D. (2006). Application of phylogenetic networks in evolutionary studies. *Mol. Biol. Evol.* 23 254–267. 10.1093/molbev/msj030 16221896

[B63] Jacobs J. H. Clark S. J. Denholm I. Goulson D. Stoate C. Osborne J. L. (2009). Pollination biology of fruit-bearing hedgerow plants and the role of flower-visiting insects in fruit-set. *Ann. Bot.* 104 1397–1404. 10.1093/aob/mcp236 19770165 PMC2778384

[B64] Jaramillo-Correa J. P. Aguirre-Planter E. Khasa D. P. Eguiarte L. E. Pinero D. Furnier G. R. (2008). Ancestry and divergence of subtropical montane forest isolates: molecular biogeography of the genus Abies (Pinaceae) in southern Mexico and Guatemala. *Mol. Ecol.* 17 2476–2490. 10.1111/j.1365-294X.2008.03762.x 18422927

[B65] Jaramillo-Correa J. P. Beaulieu J. Bedig F. T. Bousquet J. (2006). Decoupled mitochondrial and chloroplast DNA population structure reveals holocene collapse and population isolation in a threatened Mexican-endemic conifer. *Mol. Ecol.* 15 2787–2800. 10.1111/j.1365-294X.2006.02974.x 16911200

[B66] Jordano P. (2010). Pollen, seeds and genes: the movement ecology of plants. *Heredity* 105 329–330. 10.1038/hdy.2010.28 20332803

[B67] Ju L. Wang H. Jiang D. (2007). Simulation of the Last Glacial Maximum climate over East Asia with a regional climate model nested in a general circulation model. *Palaeogeogr. Palaeoclimatol. Palaeoecol.* 248 376–390. 10.1016/j.palaeo.2006.12.012

[B68] Kato M. Kosaka Y. Kawakita A. Okuyama Y. Kobayashi C. Phimminith T. (2008). Plant–pollinator interactions in tropical monsoon forests in Southeast Asia. *Am. J. Bot.* 95 1375–1394. 10.3732/ajb.0800114 21628146

[B69] Knox E. B. Palmer J. D. (1995). Chloroplast DNA variation and the recent radiation of the giant senecios (Asteraceae) on the tall mountains of eastern Africa. *Proc. Natl. Acad. Sci. U.S.A.* 92 10349–10353. 10.1073/pnas.92.22.10349 7479782 PMC40794

[B70] Krauss S. L. He T. Barrett L. G. Lamont B. B. Enright N. J. Miller B. P. (2009). Contrasting impacts of pollen and seed dispersal on spatial genetic structure in the bird-pollinated Banksia hookeriana. *Heredity* 102 274–285. 10.1038/hdy.2008.118 19002205

[B71] Ku T. C. (1985). “Rosa,” in *Flora Reipublicae Popularis Sinicae*, eds Yu T. T. Ku T. C. (Beijing: Science Press), 37.

[B72] Ku T. C. Robertson K. R. (2003). “Rosa (Rosaceae),” in *Flora of China*, eds Wu Z. Y. Raven P. H. (St. Louis, MO: Missouri Botanical Garden Press), 339–381.

[B73] Kumar S. Stecher G. Tamura K. (2016). MEGA7: molecular evolutionary genetics analysis version 7.0 for bigger datasets. *Mol. Biol. Evol.* 33 1870–1874. 10.1093/molbev/msw054 27004904 PMC8210823

[B74] Lanfear R. Frandsen P. B. Wright A. M. Senfeld T. Calcott B. (2017). PartitionFinder 2: new methods for selecting partitioned models of evolution for molecular and morphological phylogenetic analyses. *Mol. Biol. Evol.* 34 772–773. 10.1093/molbev/msw260 28013191

[B75] Librado P. Rozas J. (2009). DnaSP v5: a software for comprehensive analysis of DNA polymorphism data. *Bioinformatics* 25 1451–1452. 10.1093/bioinformatics/btp187 19346325

[B76] Link-Pérez M. A. (2010). *Revision and Molecular Systmatics of the Neotrpical Fern Genus Adiantopsis (Pteridaceae).* Doctoral Dissertation, Miami University, Miami.

[B77] Liu J. Q. (2016). The integrative species concept and species on the speciation way. *Biodivers. Sci.* 24 1004–1008. 10.17520/biods.2016222 23869749

[B78] Liu J.-Q. Wang Y.-J. Wang A.-L. Hideaki O. Abbott R. J. (2006). Radiation and diversifcation within the *Ligularia*–*Cremanthodium*–*Parasenecio* complex (Asteraceae) triggered by uplift of the Qinghai-Tibetan Plateau. *Mol. Phylogenet. Evol.* 38 31–49. 10.1016/j.ympev.2005.09.010 16290033

[B79] Liu Y. P. Ren Z. Harris A. J. Peterson P. M. Wen J. Su X. (2018). Phylogeography of Orinus (Poaceae), a dominant grass genus on the Qinghai-Tibet Plateau. *Bot. J. Linnean Soc.* 186 202–223. 10.1093/botlinnean/box091

[B80] Mannion P. D. Upchurch P. Benson R. B. Goswami A. (2014). The latitudinal biodiversity gradient through deep time. *Trends Ecol. Evol.* 29 42–50. 10.1016/j.tree.2013.09.012 24139126

[B81] Marsico T. D. Hellmann J. J. Romero-Severson J. (2009). Patterns of seed dispersal and pollen flow in *Quercus garryana* (Fagaceae) following post-glacial climatic changes. *J. Biogeogr.* 36 929–941. 10.1111/j.1365-2699.2008.02049.x

[B82] Mayr E. (1942). *Systematics and the Origin of Species, From the Viewpoint of a Zoologist.* New York, NY: Columbia University Press.

[B83] Miller M. A. Pfeiffer W. Schwartz T. (2010). “Creating the CIPRES science gateway for inference of large phylogenetic trees,” in *Proceedings of the Gateway Computing Environments Workshop (GCE)*, (New Orleans), 1–8. 10.1109/GCE.2010.5676129

[B84] Myers N. Mittermeier R. A. Mittermeier C. G. Da Fonseca G. A. Kent J. (2000). Biodiversity hotspots for conservation priorities. *Nature* 403 853–858. 10.1038/35002501 10706275

[B85] Nason J. D. Herre E. A. Hamrick J. L. (1996). Paternity analysis of the breeding structure of strangler fig populations: evidence for substantial long-distance wasp dispersal. *J. Biogeogr.* 23 501–512. 10.1111/j.1365-2699.1996.tb00012.x

[B86] Nei M. (1987). *Molecular Evolutionary Genetics.* New York, NY: Columbia University Press.

[B87] Nosil P. (2008). Speciation with gene flow could be common. *Mol. Ecol.* 17 2103–2106. 10.1111/j.1365-294X.2008.03715.x 18410295

[B88] Peakall R. Smouse P. E. (2012). GenAlEx 6.5: genetic analysis in Excel. Population genetic software for teaching and research–an update. *Bioinformatics* 28 2537–2539. 10.1093/bioinformatics/bts460 22820204 PMC3463245

[B89] Phillips S. J. Anderson R. P. Schapire R. E. (2006). Maximum entropy modelling of species’ geographic distributions. *Ecol. Model.* 190 231–259. 10.1371/journal.pone.0187602 29190296 PMC5708625

[B90] Pons O. Petit R. J. (1996). Measuring and testing genetic differentiation with ordered versus unordered alleles. *Genetics* 144 1237–1245. 8913764 10.1093/genetics/144.3.1237 PMC1207615

[B91] Pritcharda J. K. Wena X. Falush D. (2009). *Documentation for Structure Software: Version 2.3. [Internet].* Available at: http://pritch.bsd.uchicago.edu/structure_software/release_versions/v2.3.2/structure_doc.pdf.2009

[B92] Provan J. Bennett K. D. (2008). Phylogeographic insights into cryptic glacial refugia. *Trends Ecol. Evol.* 23 564–571. 10.1016/j.tree.2008.06.010 18722689

[B93] Qian H. Ricklefs R. E. (2000). Large-scale processes and the Asian bias in species diversity of temperate plants. *Nature* 407 180–182. 10.1038/35025052 11001054

[B94] Qian H. Sandel B. (2017). Phylogenetic structure of regional angiosperm assemblages across latitudinal and climatic gradients in North America. *Glob. Ecol. Biogeogr.* 26 1258–1269. 10.1111/geb.12634

[B95] Qiu Y. X. Fu C. X. Comes H. P. (2011). Plant molecular phylogeography in China and adjacent regions, tracing the genetic imprints of quaternary climate and environmental change in the world’s most diverse temperate flora. *Mol. Phylogenet. Evol.* 59 225–244. 10.1016/j.ympev.2011.01.012 21292014

[B96] Rainey P. B. Travisano M. (1998). Adaptive radiation in a heterogeneous environment. *Nature* 394 69–72. 10.1038/27900 9665128

[B97] Raxworthy C. J. Ingram C. M. Rabibisoa N. Pearson R. G. (2007). Applications of ecological niche modeling for species delimitation: a review and empirical evaluation using day geckos (Phelsuma) from Madagascar. *Syst. Biol.* 56 907–923. 10.1080/10635150701775111 18066927

[B98] Raxworthy C. J. Martinez-Meyer E. Horning N. Nussbaum R. A. Schneider G. E. Ortega-Huerta M. A. (2003). Predicting distributions of known and unknown reptile species in Madagascar. *Nature* 426 837–841. 10.1038/nature02205 14685238

[B99] Raymond M. Rousset F. (1995). Genepop (version-1.2) population-genetics software for exact tests and ecumenicism. *J. Heredity* 86 248–249. 10.1093/oxfordjournals.jhered.a111573

[B100] Ridley M. (2004). *Evolution*, Third Edn Malden, MA: Blackwell Publishing.

[B101] Ritz C. M. Kohnen I. Groth M. Theissen G. Wissemann V. (2011). To be or not to be the odd one out–allele-specific transcription in pentaploid dogroses (Rosa L. sect. Caninae (DC.) Ser). *BMC Plant Biol.* 11:37. 10.1186/1471-2229-11-37 21345190 PMC3053229

[B102] Sage R. D. Selander R. K. (1975). Trophic radiation through polymorphism in cichlid fishes. *Proc. Natl. Acad. Sci. U.S.A.* 72 4669–4673. 10.1073/pnas.72.11.4669 16592286 PMC388785

[B103] Salisbury E. J. (1942). *The Reproductive Capacity of Plants.* London: G. Bell.

[B104] Sanders N. J. Rahbek C. (2012). The patterns and causes of elevational diversity gradients. *Ecography* 35 1–3. 10.1111/j.1600-0587.2011.07338.x

[B105] Schluter D. (2001). Ecology and the origin of species. *Trends Ecol. Evol.* 16 372–380. 10.1016/S0169-5347(01)02198-X 11403870

[B106] Schoener T. W. (1968). The anolis lizards of bimini: resource partitioning in a complex fauna. *Ecology* 49 704–726. 10.2307/1935534

[B107] Schulke B. Waser N. M. (2001). Long-distance pollinator flights and pollen dispersal between populations of *Delphinium nuttallianum*. *Oecologia* 127 239–245. 10.1007/s004420000586 24577655

[B108] Stamatakis A. (2014). RAxML version 8: a tool for phylogenetic analysis and post-analysis of large phylogenies. *Bioinformatics* 30 1312–1313. 10.1093/bioinformatics/btu033 24451623 PMC3998144

[B109] Stephens M. Donnelly P. (2003). A comparison of bayesian methods for haplotype reconstruction from population genotype data. *Am. J. Hum. Genet.* 73 1162–1169. 10.1086/379378 14574645 PMC1180495

[B110] Stephens M. Smith N. J. Donnelly P. (2001). A new statistical method for haplotype reconstruction from population data. *Am. J. Hum. Genet.* 68 978–989. 10.1086/319501 11254454 PMC1275651

[B111] Stibig H.-J. Achard F. Fritz S. (2004). A new forest cover map of continental southeast Asia derived from SPOT-VEGETATION satellite imagery. *Appl. Veg. Sci.* 7 153–162. 10.1111/j.1654-109X.2004.tb00606.x

[B112] Stuart B. L. Inger R. F. Voris H. K. (2006). High level of cryptic species diversity revealed by sympatric lineages of Southeast Asian forest frogs. *Biol. Lett.* 2 470–474. 10.1098/rsbl.2006.0505 17148433 PMC1686201

[B113] Swofford D. L. (2003). *PAUP^∗^ Phylogenetic Analysis Using Parsimony (and Other Methods).* Sunderland, MA: Sinauer Associates.

[B114] Tamura K. Dudley J. Nei M. Kumar S. (2007). MEGA4, Molecular Evolutionary Genetics Analysis (MEGA) software version 4.0. *Mol. Biol. Evol.* 24 1596–1599. 10.1093/molbev/msm092 17488738

[B115] Thompson J. D. Gibson T. J. Plewniak F. Jeanmougin F. Higgins D. G. (1997). The CLUSTAL_X windows interface: flexible strategies for multiple sequence alignment aided by quality analysis tools. *Nucleic Acids Res.* 25 4876–4882. 10.1093/nar/25.24.4876 9396791 PMC147148

[B116] Thompson J. D. Higgins D. G. Gibson T. J. (1994). CLUSTAL W: improving the sensitivity of progressive multiple sequence alignment through sequence weighting, position-specific gap penalties and weight matrix choice. *Nucleic Acids Res.* 22 4673–4680. 10.1093/nar/22.22.4673 7984417 PMC308517

[B117] von Humboldt A. (1807). *Essai Sur Sur La Géographie Des Plantes.* Chicago: University of Chicago Press.

[B118] Warren D. L. Glor R. E. Turelli M. (2008). Environmental niche equivalency versus conservatism: quantitative approaches to niche evolution. *Evolution* 62 2868–2883. 10.1111/j.1558-5646.2008.00482.x 18752605

[B119] Warren D. L. Glor R. E. Turelli M. (2010). ENMTools: a toolbox for comparative studies of environmental niche models. *Ecography* 33 607–611. 10.1111/j.1600-0587.2009.06142.x

[B120] Watson H. C. Spottiswoode A. (1835). *Remarks on the Geographical Distribution of British Plants: Chiefly in Connection with Latitude, Elevation, and Climate.* London: Longman 10.5962/bhl.title.59223

[B121] Weir B. S. Cockerham C. C. (1984). Estimating F-statistics for the analysis of population structure. *Evolution* 38 1358–1370. 28563791 10.1111/j.1558-5646.1984.tb05657.x

[B122] Wen J. (1999). Evolution of the eastern Asian and eastern North American disjunct distributions in flowering plants. *Annu. Rev. Ecol. Syst.* 30 421–455. 10.1016/j.ympev.2012.05.003 22580463

[B123] Wen J. (2001). Evolution of eastern Asian–eastern North American biogeographic disjunctions: a few additional issues. *Int. J. Plant Sci.* 162 S117–S122. 10.1086/322940

[B124] Wharton P. Hine B. Justice D. (2005). *The Jade Garden: New & Notable Plants from Asia.* Portland: Timber Press.

[B125] Williams D. Dunkerley D. DeDeckker P. Kershaw P. Chappell M. (1998). *Quaternary Environments.* London: Arnold.

[B126] Willig M. R. Kaufman D. M. Stevens R. D. (2003). Latitudinal gradients of biodiversity: pattern, process, scale, and synthesis. *Annu. Rev. Ecol. Evol. Syst.* 34 273–309. 10.1146/annurev.ecolsys.34.012103.144032

[B127] Wilson E. O. (1988). “The current state of biological diversity,” in *Biodiversity*, eds Wilson E. O. Peter F. M. (Washington, DC: The National Academies Press), 521. 25032475

[B128] Xing Y. Ree R. H. (2017). Uplift-driven diversification in the Hengduan Mountains, a temperate biodiversity hotspot. *Proc. Natl. Acad. Sci. U.S.A.* 114 E3444–E3451. 10.1073/pnas.1616063114 28373546 PMC5410793

[B129] Yuan S. L. Lin L. K. Oshida T. (2006). Phylogeography of the mole-shrew (*Anourosorex yamashinai*) in Taiwan, implications of interglacial refugia in a high elevation small mammal. *Mol. Ecol.* 15 2119–2130. 10.1111/j.1365-294X.2006.02875.x 16780429

[B130] Zhao J.-L. Gugger P. F. Xia Y.-M. Li Q.-J. (2016). Ecological divergence of two closely related *Roscoea* species associated with late quaternary climate change. *J. Biogeogr.* 43 1990–2001. 10.1111/jbi.12809 21438932

