# Supplementary material for: Species Boundaries and Parapatric Speciation in the Complex of Alpine Shrubs, Rosa sericea (Rosaceae), Based on Population Genetics and Ecological Tolerances
Source: Front Plant Sci. 2019 Mar 18;10:321. doi: 10.3389/fpls.2019.00321 (PMC6432857; doi:10.3389/fpls.2019.00321)

**Table S1** Genetic diversity parameters for all 42 populations of *Rosa sericea* and *R. omeiensis* for chloroplast and nuclear LEAFY.

|  | *H_S_* | *H_T_* | *N_ST_* | *G_ST_* | permutation test (*P*) |
| --- | --- | --- | --- | --- | --- |
| chloroplast | 0.241 (0.0397) | 0.790 (0.0520) | 0.811 (0.0524) | 0.695 (0.0479) | *N_ST_* > *G_ST_* (*P* <0.01) |
| LEAFY | 0.520 (0.0348) | 0.950 (0.0083) | 0.798 (0.0372) | 0.452 (0.0363) | *N_ST_* > *G_ST_* (*P* < 0.01) |

*H_S_*, average genetic diversity within populations; *H_T_*, total genetic diversity; *N_ST_*, inter-population differentiation taking into account sequence difference; *G_ST_*, inter-population differentiation.

**Table S2** Mean estimates of genetic diversity and differentiation at eight nuclear microsatellite loci surveyed across all populations

| Locus | *N*_A_ | *H*_O_ | *H*_S_ | *H*_T_ | *F*_ST_ |
| --- | --- | --- | --- | --- | --- |
| EC587106 | 13 | 0.366 | 0.411 | 0.698 | 0.411 |
| EC586919 | 23 | 0.239 | 0.449 | 0.698 | 0.357 |
| BQ106227 | 17 | 0.374 | 0.492 | 0.745 | 0.340 |
| EC587073 | 23 | 0.492 | 0.634 | 0.753 | 0.158 |
| CF349346 | 36 | 0.478 | 0.744 | 0.925 | 0.196 |
| EC587962 | 19 | 0.567 | 0.623 | 0.832 | 0.251 |
| EC587071 | 28 | 0.677 | 0.730 | 0.905 | 0.193 |
| EC587517 | 27 | 0.501 | 0.540 | 0.890 | 0.394 |
| mean | 23.25 | 0.462 | 0.578 | 0.806 | 0.283 |

*N*_A_, observed allele number; *H*_O_, observed within-population heterozygosity; *H*_S_, expected within-population (gene) diversity; *H*_T_, overall gene diversity; *F*_ST_, among-population differentiation.

| **Table S3.** Hierarchical analysis of molecular variation (AMOVA) based on data from phased LEAFY for all 42 populations of *Rosa sericea* and *R. omeiensis* separated by taxa. | | | | | |
| --- | --- | --- | --- | --- | --- |
| Source of variation | d.f. | SS | VC | PV | *F*- statistics |
| Among groups | 1 | 10.703 | 0.02472 | 5.05 | FCT=0.05046* |
| Among populations within groups | 40 | 157.718 | 0.20691 | 42.24 | FSC=0.44480** |
| Within populations | 710 | 183.369 | 0.25827 | 52.72 | FST=0.47282** |
| Total | 751 | 351.790 | 0.48990 |  |  |
| d.f., degree of freedom; SS, sum of squares; VC, variance components; PV, percentage of variation | | | | | |
| **P<0.0001; *0.001<P<0.005 (based on 1023 permutations) | | | | | |

| **Table S4.** Hierarchical analysis of molecular variation (AMOVA) based on data from the three chloroplast DNA spacers for all 42 populations of *Rosa sericea* and *R. omeiensis* separated by taxa. | | | | | |
| --- | --- | --- | --- | --- | --- |
| Source of variation | d.f. | SS | VC | PV | *F*- statistics |
| Among groups | 1 | 10.399 | 0.04855 | 11.52 | FCT=0.11518* |
| Among populations within groups | 40 | 122.177 | 0.27041 | 64.16 | FSC=0.72508** |
| Within populations | 417 | 42.753 | 0.10253 | 24.33 | FST=0.75675** |
| Total | 458 | 175.329 | 0.42148 |  |  |
| d.f., degree of freedom; SS, sum of squares; VC, variance components; PV, percentage of variation | | | | | |
| **P<0.0001; *0.001<P<0.01 (based on 1023 permutations) | | | | | |

**Figure S1.** Phylogenetic topography based on plastid DNA dataset using Neighbor-joining method. Bootstrap values (BP) are given and topology built by Maximum Likelihood (ML) is not shown since no significant discordance were found compared to the NJ method.





**Figure S2.** Delta K values calculated by Evanno’s (2005) method based on variation at eight nuclear microsatellite (**nSSR**) loci.

**Figure S3.** Results of a principal component analysis (PCA) using environmental variables from all occurrence data points. A) the result of 19 bioclimatic variables and B) the monthly maximum and minimum temperatures. Elevation gradient also included to show the altitude perfection of each species.


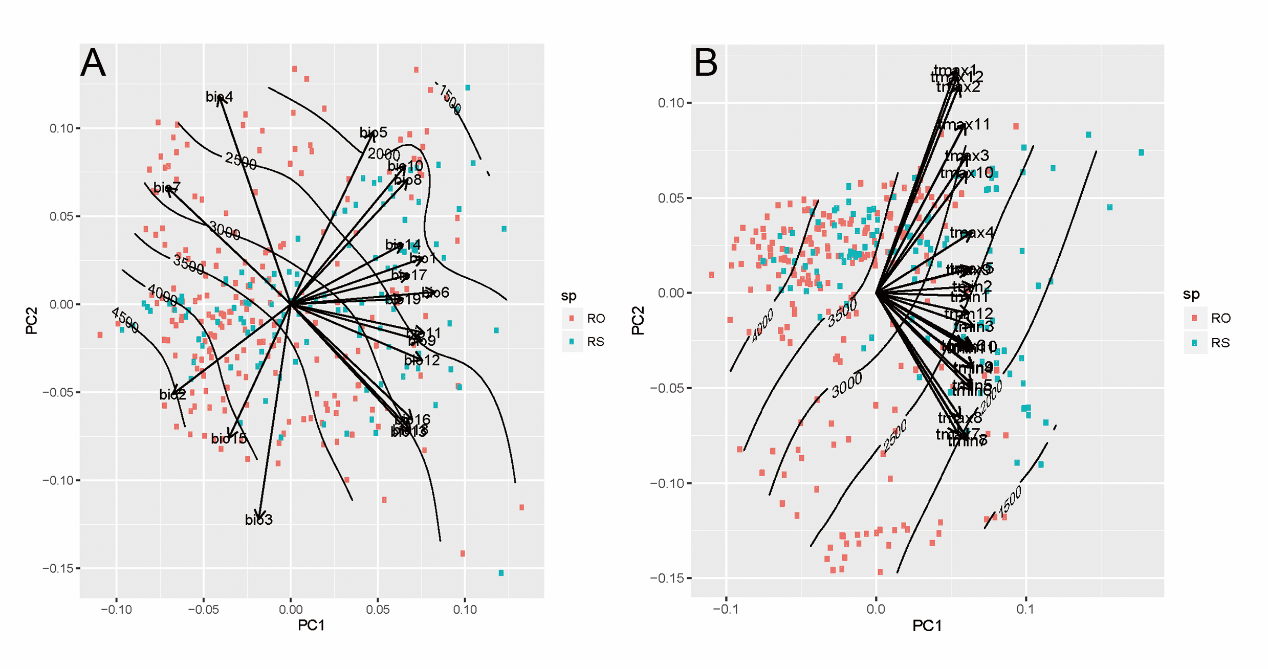

Supplement: Supplementary file 1 [file Data_Sheet_1.docx]
